# Maackiain Reduces Neuroinflammation by Modulating Inflammatory Signals in LPS-Induced *In Vitro* and *In Vivo* Models

**DOI:** 10.4014/jmb.2508.08046

**Published:** 2026-02-05

**Authors:** Tianchan Yun, Yue Xiao, Yanmei Gong, Yanxian Lai, Shiya Huang, Yanqing Ma, Yixi Zeng, Lanyue Zhang, Cong Deng

**Affiliations:** 1 Guangzhou First People’s Hospital, School of Medicine, South China University of Technology, No.1 Panfu Road, Guangzhou 510180, P. R. China; 2 School of Biomedical and Pharmaceutical Sciences, Guangdong University of Technology, Guangzhou 510006, P. R. China

**Keywords:** Neuroinflammation, Maackiain, Lipopolysaccharide, BV2, Inflammation

## Abstract

Neuroinflammation, an immune process in the central nervous system (CNS), is a key contributor to a range of neurological diseases, including neurodegenerative disorders (*e.g.*, Alzheimer’s and Parkinson’s disease), stroke, and depression, underscoring its significant pathological relevance. While maackiain (MAA) exhibits potent anti-inflammatory activity, its potential to mitigate neuroinflammation remains poorly understood. This study investigated the therapeutic effects of MAA on lipopolysaccharide (LPS)-induced neuroinflammation and its underlying mechanisms. *In vitro*, MAA significantly improved BV2 cell viability and reduced nitric oxide (NO) expression in LPS-treated cells, decreased the expression of reactive oxygen species (ROS), and it also inhibited the accumulation of Ferrous ion (Fe^2+^) and lipid peroxides as well as the damage to mitochondria. Higher concentrations of MAA were more effective, consistent with subsequent animal experiments. *In vivo*, mice treated with MAA showed improved memory in the Morris water maze compared to the LPS group. Nissl staining revealed fewer IBA-1 positive cells and a decrease in COX-2 and IL-6 levels in the hippocampus and cortex. This compound also increased the number of normal neurons in the cortex and CA3 region. The results of this study highlight the inhibitory effects of MAA on neuroinflammation, suggesting its potential as an effective therapeutic agent for treating neuroinflammation.

## Introduction

Neuroinflammation is a condition caused by autoimmune, innate immune dysfunction, or infection of the nervous system [1] and is manifested by an increase in the production and release of inflammatory mediators such as reactive oxygen species (ROS), IL-6, and COX-2 by various immune cells (such as microglia cells) [2]. Studies have shown that neuroinflammation is closely related to a variety of neurodegenerative and neuropsychiatric diseases such as Alzheimer's disease (AD), Parkinson's disease, and multiple sclerosis (MS) [3]. Therefore, the treatment or inhibition of neuroinflammation plays a key role in the treatment of a variety of neurological diseases. Current therapeutic approaches, however, are beset by major challenges. Conventional anti-inflammatory agents like Non-Steroidal Anti-Inflammatory Drug have shown limited clinical benefit, hampered by poor blood-brain barrier (BBB) penetration, off-target effects, and considerable systemic toxicity. While novel microglia-targeting therapies, such as the Receptor for Advanced Glycation End Products antagonist TTP488 [4], represent a promising avenue for disease modification, they are still in early development, and their long-term efficacy, safety, and accessibility remain unproven [5]. Consequently, identifying more accessible, safer, and mechanistically clear anti-neuroinflammatory drugs is a pressing priority in the field.

Nitric oxide (NO) plays a pivotal role in neuronal functional and structural impairment, serving as a direct pathway through which neuroinflammation induces neuronal cell death [6]. Concurrently, BV2 microglia respond to damage-associated molecular patterns (DAMPs) by generating ROS. Given that oxidative stress is a hallmark of AD, characterized by mitochondrial DNA damage and lipid peroxidation, understanding how BV2 cells become dysregulated and contribute to oxidative stress via ROS production is crucial for comprehending inflammation-associated neurodegeneration [7]. Furthermore, the phenomenon of ferroptosis is linked to the promotion of inflammation, and its inhibitors exhibit both antioxidant and anti-inflammatory properties. Ferroptosis is defined by elevated levels of ROS and Fe^2+^, and this mode of cell death is associated with various neurological disorders; studies have shown that LPS can induce Fe^2+^ accumulation and ferroptosis in the liver [8]. Mitochondria-derived DAMPs are recognized by immune receptors on microglia, which in turn exacerbates neuroinflammation [9]. Ultimately, neuroinflammation is a core pathological mechanism underlying cognitive and memory deficits, playing a key role in neurodegenerative diseases and postoperative cognitive dysfunction, where neuronal injury leads to impaired short-term memory [10].

Natural products hold great promise for the treatment of neuroinflammation. The neuroprotective properties of natural compounds and their metabolites have been extensively investigated and reported in the literature for the management of neurodegenerative diseases (NDs) [11]. Notably, the natural product monomer MAA has been reported to inhibit human monoamine oxidase B (MAO-B), which is used in the treatment of diseases such as Alzheimer's disease, Parkinson's disease, and depression [12]. MAA has also been found to have Anti-inflammatory [13], anti-allergy [14], anti-tumor [15] and pro-apoptotic [16] functions. In contrast, there is less research on anti-inflammatories. Based on the anti-inflammatory and neuroprotective potential of MAA, the present study aimed to investigate the effects and mechanisms of MAA on LPS-induced neuroinflammation.

## Materials and Methods

### Materials

Maackiain (MAA, C_16_H_12_O_5_, ≥95% purity, molecular weight: 284.26 g/mol) was purchased from Chengdu Mede Biotechnology Co., LTD. (China). TTP488 (Azeliragon), DAPI fluorescence labeling solution, and lipopolysaccharide (LPS) were purchased from MEDMOL (China). Ionized calcium binding adaptor molecule 1 (IBA-1), interleukin-6 (IL-6), and cyclooxygenase-2 (COX-2) antibodies were obtained from Yiyan (China). Nissl staining solution (toluidine blue staining technique) and the anti-tumor necrosis factor polyclonal antibody were sourced from Tengyue Biotechnology Co., Ltd. (China). Phosphate-buffered saline (PBS), penicillin-streptomycin (PS) solution, trypsin, Dulbecco's modified Eagle medium (DMEM), methyl-thiazolyl-tetrazolium (MTT), and fetal bovine serum (FBS) were sourced from Yisheng Biotechnology (China). The NO kit was acquired from Beyotime Biotechnology (China). The BV2 cell line was obtained from the China Center for Type Culture Collection (China).

### Instrumentation

CO_2_ incubator (Thermo Fisher Scientific, Qisheng Medical Equipment Co., Ltd., China). Microplate reader (Shandong Hengmei Electronic Technology Co., LTD., China). Z1 automatic digital slide scanner from Carl Zeiss (Germany).

### Cell Culture and Processing

The powder of MAA (molecular weight: 284.26 g/mol) was dissolved in a DMSO solution to obtain a 50 mM stock solution. The solution was subjected to filtration using a microporous membrane with a pore size of 0.22 μm and aseptically stored in a refrigerator at 4°C. The complete medium was prepared according to the ratio of 89% DMEM basal medium, 10% FBS, and 1% PS. The cells were grown in T25 culture flasks. BV2 cells were transferred to 4 ml of previously configured medium and incubated in a CO_2_ incubator at a constant temperature of 37°C and 5% CO_2_. The nutrient solution was replaced daily. Upon reaching confluence, BV2 cells were detached with 1 ml of trypsin solution and incubated for 1 min. After complete digestion, the solution was centrifuged, the trypsin was discarded, and complete medium was added to resuspend and mix the cells evenly. The cells were then added to a 96-well plate, and the cell concentration per well was diluted to 50,000 cells/well [15, 16]. The 1 mg/ml LPS solution was diluted to 10 μg/ml and added to the wells to achieve a final concentration of 100 ng/ml. After culturing for 2 h, drugs of different concentrations were added. The BV2 cells were then placed back into the incubator and cultured for 24 h under the previously established conditions.

### Cell Viability Assay

The inhibition rate of cell proliferation could be measured by the MTT assay [17]. BV2 cells with cell confluence above 90% were blown down by trypsin digestion, cell concentration was adjusted to 1 × 10^6^ cells /ml, and the diluted cell suspension was added to a 96-well plate (1 × 10^5^ cells /well) to detect the cytotoxicity of MAA at different concentrations. The well plates of the inoculated cells were placed in an incubator and cultured for 24 h. The incubation was completed, the medium was carefully discarded, and 1 mg/ml LPS solution was diluted to 10 μg/ml and then added to each well, except for the control group, resulting in a final LPS concentration of 0.1 μg/ml per empty, after 2 h of incubation. Each was well treated with different concentrations of MAA (0, 2.5, 5, 10, 20, 40 μM). At the same time, the zero-setting group and the LPS group were set up. After 24 h, remove the old medium with care, and 100 μl of MTT 0.5 mg/ml was added to each well. After incubation in the incubator for 4 h, the MTT was meticulously removed, and then 100 μl of DMSO was introduced into each well. The 96-well plate was placed on a shaker for 10 min to completely dissolve Formazan, and absorbance was then measured at a wavelength of 570 nm using a microplate reader [18]. Cell viability (%) = (OD with drug - OD blank) / (OD control - OD blank) × 100%.[Fig F1]

### Measurement of NO Production

The BV2 cells with a confluence degree of more than 90% were digested and detached using trypsin and resuspended in a complete medium. The cell suspension was grown in 96-well plates under the same conditions and procedures as those used for the MTT assay. After culturing for 24 h, the medium was discarded. The diluted 1 mg/ml LPS solution was adjusted to 10 μg/ml, and then added to each well to achieve a final concentration of 0.1 μg/ml. After culturing for 2 h, 100 μl of 0, 2.5, 5, 10, 20, and 40 μM MAA was added to each well, respectively. At the same time, an LPS group and a blank group were established. After 24 h, 50 μl of cell supernatant was extracted from each well and transferred to another unused plate. Following the instructions of the NO kit, the absorbance value at a wavelength of 562 nm was determined.

### Detection of ROS Assays

In this experiment, 2',7'-dichlorodihydrofluorescein diacetate (DCFH-DA) was used as a fluorescent probe to detect the level of intracellular ROS. The methods for cell culture, drug treatment, and model establishment are consistent with those described earlier. The experimental procedure is as follows: After removing the 24-well plate containing BV2 cells from the 37°C, 5% CO_2_ incubator, discard the culture medium and wash each well three times with PBS for 5 min each, a 10 μM DCFH-DA probe diluted with PBS was added, and the cells were incubated in an incubator at 37°C and 5% CO_2_ for 60 min. Then, the cells were washed three times with PBS for 5 min each time. Finally, the cells were observed under an inverted fluorescence microscope in a dark environment with an excitation wavelength of 488 nm. Three random fields of view were selected from each well and imaged, and then the optical density values in the images were analyzed using Image-Pro Plus 6.0 software (Media Cybernetics,USA)

### Detection of Fe^2+^ Levels

FerroOrange is a fluorescent probe specifically designed for detecting Fe^2+^. Its principle is based on a significant increase in fluorescence intensity upon binding with Fe^2+^. FerroOrange itself exhibits weak fluorescence, but when bound to Fe^2+^, it forms a highly fluorescent complex that emits intense orange fluorescence. By measuring changes in fluorescence intensity, Fe^2+^ levels in cells can be quantitatively analyzed. This method is widely applied in iron metabolism research and the detection of iron-dependent cell death (ferroptosis). The methods for cell culture, drug treatment, and model establishment are consistent with those described earlier. The experimental procedure is as follows: After removing the 24-well plate containing BV2 cells from the 37°C, 5% CO_2_ incubator, discard the culture medium and wash each well three times with PBS for 5 min each. Subsequently, add 1 ml of 1 μM FerroOrange working solution (diluted in PBS buffer) to each well and incubate the plate in the 37°C, 5% CO_2_ incubator for 30 min. Following incubation, observe the cells under an inverted fluorescence microscope in a dark environment. Randomly select three fields of view per well, capture images, and analyze the fluorescence intensity using Image Pro Plus software.

### Detection of Lipid Peroxidation Level

BODIPY 581/591 C_11_ (Shanghai DONGREN CHEMICAL Technology Co., LTD, CN) is a fluorescence-based lipid peroxidation detection probe, and its detection mechanism relies on the wavelength shift of the fluorescence signal. The probe itself does not directly react with lipid peroxides but can specifically react with reactive intermediates, such as lipid radicals generated during lipid peroxidation, enabling dynamic monitoring of intracellular lipid peroxidation levels. This fluorescent probe usually emits red fluorescence, and as lipid peroxidation proceeds, the fluorescence changes from red to green. By tracking changes in the fluorescence signal (from red to green), lipid peroxidation can be monitored in real time. It features high selectivity and sensitivity and is widely used in ferroptosis research and lipid peroxidation detection.

The methods for cell culture, drug addition, and model establishment are the same as described previously. The experiment is carried out as follows: Take out the 24-well plate with BV2 cells from the incubator at 37°C with 5% CO_2_, discard the culture medium, and wash each well three times with PBS for 5 min each time. Subsequently, dilute the working solution of the lipid peroxidation fluorescent probe C_11_ BODIPY 581/591 to 50 μM with PBS buffer, and add 1 ml of the working solution to each well of the 24-well plate. Incubate the cells at 37°C in the dark for 30 min, then wash the cells three times with PBS buffer for 5 min each time. Finally, observe the cells using an inverted fluorescence microscope in a dark environment, randomly select 3 fields of view per well, and capture images. Calculate the areas of the green and red fluorescence regions using Image Pro Plus software, and further calculate the green/red fluorescence ratio for each group.

### Mitochondrial Membrane Potential Assay

The mitochondrial membrane potential (ΔΨm) of BV2 cells was assessed using JC-10 fluorescent dye (Jiangsu Addison Biotechnology Co. LTD, CN), which exhibits potential-dependent fluorescence characteristics. At high membrane potentials, JC-10 forms aggregates emitting red fluorescence (590 nm), while at depolarized potentials, it remains monomeric with green fluorescence emission (530 nm). Cells at >90% confluency were trypsinized, adjusted to 1 × 10^5^ cells/ml in complete medium, and seeded in 24-well plates (1 ml/well). Following experimental treatments, cells were washed with PBS and incubated with 10 μM JC-10 in 20 mM HEPES buffer (pH 7.4) at 37°C with 5% CO_2_ for 20 min. After washing with ice-cold HEPES buffer and replacing it with complete medium, fluorescence imaging was performed using an automated live-cell imaging system. Three random fields per well were captured, and Image Pro Plus 6.0 software was used to quantify JC-10 aggregate (red) and monomer (green) fluorescence intensities, with the red/green fluorescence ratio calculated as an indicator of ΔΨm integrity. This method enables sensitive detection of early apoptotic changes through quantitative assessment of mitochondrial depolarization.

### Animals and Experimental Design

**Fig. 2:**
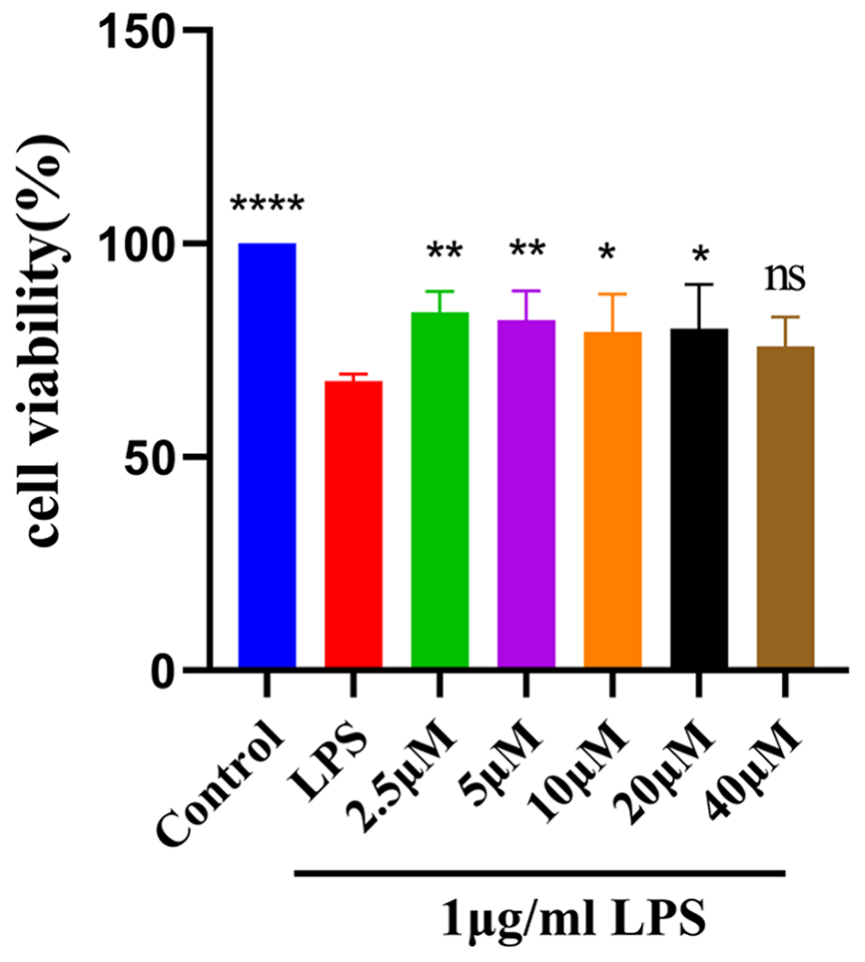
Maackiain pretreatment attenuated LPS-induced BV2 cell death. (vs LPS group, ns (not significant difference), *p < 0.05, **p < 0.01, ***p < 0.0001, and ****p < 0.0001). Data are presented as mean ± SD.

SPF-grade male C57BL/6 mice, aged 7 to 8 weeks and weighing between 18 and 22 grams, were acquired from Guangzhou Ruige Biology Co., Ltd. (Laboratory Animal Permit Number: SCXK (Guangdong) 2021-0059). After one week of adaptive feeding, 50 C57BL/6 mice were assigned to five different groups: (1) Control group (administered normal saline); (2) LPS model group; (3) LPS + TTP488 group (5 mg/kg); (4) LPS + MAA-L group (low-dose MAA, 25 mg/kg); (5) LPS + MAA-H group (high-dose MAA, 50 mg/kg). All drugs were prepared in 0.9% saline and sonicated to ensure uniform suspension. The experimental procedure and administration schedule are illustrated in Fig. 2. The Control group received an intraperitoneal (i.p.) injection of 0.9% saline. The LPS group was administered an i.p. injection of LPS at 250 μg/kg (Control and LPS groups also received oral gavage of 0.9% saline (0.2 ml/10 g) to match the vehicle volume). The TTP488 (5 mg/kg) and MAA groups were co-administered their respective compound via oral gavage and an i.p. injection of LPS. All treatments were administered once daily for 14 consecutive days. The injection volume for the Control and LPS groups was calculated at 0.1 ml per 10 g of body weight, based on each mouse’s pre-dosing weight. The oral gavage volume for the TTP488 and MAA groups was calculated at 0.2 ml per 10 g of body weight.

### Morris Water Maze Test (MWM)

The duration of the MWM experiment was 7 days. Days 1 to 6 involved positioning navigation tasks, while day 7 was dedicated to spatial exploration. The location navigation experiment was divided into a training stage and a test stage. (1) Training stage: from day 1 to day 5, mice in all groups were gently put into the water from the same quadrant and facing the pool wall, and video tracking began. The computer system recorded the duration from the moment of entering the water to the point of locating the survival platform and the behavioral trace, which was the Escape Latency. If the mice can locate the survival platform in less than a minute, the video system stops automatic recording and puts the mice on the platform for 10 sec to make them remember the surrounding environment. If the mice found the survival platform for more than 1 min, the video recording system automatically stopped recording after 1 min, and we put the mice on the platform for 10sec to make them remember the surrounding environment. After training in one quadrant, mice in all groups were placed in a different quadrant four times a day; each quadrant was different, and the order of quadrants was different each day. (2) Test phase: D6, the escape latency of mice entering the water from different quadrants was recorded, the room was kept quiet, and people were avoided. At this stage, no matter whether the mice could find the survival platform within 1 minute or not, the mice did not need to stay on the platform. Space exploration experiment: D7, the survival platform was removed, and the mice were placed into the water from the diagonal quadrant of the specific quadrant in which the original survival platform was situated. The computer system recorded the Times and action traces of the mice entering the original survival platform quadrant within 1 min [19].

### Fluorescent Immunoassay

Upon completion of the MWM test, the whole brains of the sacrificed mice were retained, and a portion of the hippocampus was isolated. The isolated animal tissues were preserved using 4% paraformaldehyde and subsequently sectioned into 4-micrometer-thick paraffin slices. The slides were placed in a citrate antigen retrieval solution contained within a repair box and heated in a microwave for 8 min to bring them to a boil. The heat was paused for 8 min to maintain warmth, followed by 7 min at medium-low temperature. After natural cooling, PBS (pH 7.4) was used to wash the slides three times using a decolorization shaker (5 min each time). Overnight incubation was performed on the tissue at 4°C with an IBA-1 antibody (Jiangsu Affinity Company, CN) at a dilution ratio of 1:60. The sections were then washed and incubated for one h at 25°C with a secondary antibody (1:800 dilution) while being protected from light. Ultimately, the sections were subjected to staining with DAPI dye at a dilution of 1:100 for a duration of 10 min. Images were captured using a consistent exposure time and threshold setting across all groups to ensure comparable fluorescence intensity measurements.

### Nissl Staining Experiments

PBS was used to wash the brain tissue samples twice (5-10 min each). After paraffin embedding, 5 μm sections were made, followed by centrifugation. For dewaxing, the brain tissue sections were sequentially soaked in xylene I and II for a duration of 30 min each until they became transparent. Afterward, the specimens were soaked in absolute ethanol solutions I and II. for 10 min each, and this was followed by 5-min immersions in 95%, 90%, 85%, and 70% ethanol. The sections were subsequently rinsed twice using distilled water for a duration of 2 min each to rehydrate. Toluidine blue staining: Brain tissue sections were stained for 10 min and washed twice with water for 2 min each time. Next, the sections were successively dehydrated in 70%, 85%, and 95% ethanol for 5 min each, followed by dehydration with xylene I and II for a duration of 10 min each. Finally, the slices processed by the above procedure were mounted with neutral resin, and after the resin dried, the slides were scanned using an automatic slide scanner. After exporting the images, the number of normal neurons was determined using Image Pro Plus software.

### Immunohistochemistry

Sections were incubated with a citric acid solution (pH = 6.0) for antigen repair. Mouse brain tissue sections underwent treatment with 3% hydrogen peroxide (H_2_O_2_) for a duration of 25 min under dark conditions. Next, 3% BSA was used for blocking non-specific binding for 30 min. Then, the appropriate amount of primary antibody was added and incubated overnight at 4°C. To avoid dryness of the primary antibody, the slides and wet cotton balls were boxed and incubated in a 4°C refrigerator overnight. Afterward, a secondary antibody was applied, followed by DAB for color development and hematoxylin for nuclear counterstaining. Images were acquired using (Axis Communications, Sweden) and scanned with the Z1 automatic digital slide scanner.

### Transcriptome Analysis of Brain Tissue in Morris Water Maze (MWM) Mice

Mouse brain tissues were retrieved from -80°C and transported on dry ice to Shanghai Majorbio Bio-pharm Technology Co., Ltd. All mRNA transcribed from the brain tissues was sequenced on the (Illumina NovaSeq 6000 platform, USA). Differentially expressed genes (DEGs) were screened and analyzed using DESeq2 software based on the gene expression levels across the LPS, control, TTP488, MAA-L, and MAA-H groups. The screening criteria for DEGs were set as *p* < 0.05 and |log2FC| ≥ 0.263 (equivalent to a Fold Change, FC, of ≥ 1.2 or ≤ 0.83), where FC represents the magnitude of up- or down-regulation in gene expression between samples. Gene Ontology (GO) and Kyoto Encyclopedia of Genes and Genomes (KEGG) enrichment analyses were subsequently performed on the identified DEGs. Based on Fisher's exact test and the hypergeometric distribution, *p*-values were calculated, and GO and KEGG terms with *p* < 0.05 were considered significantly enriched.

### Network Pharmacology

To explore the potential connections between MAA and neuroinflammation-related processes, a series of bioinformatics analyses was conducted. First, the GeneCards database was queried using keywords associated with LPS-induced neuroinflammation. The SMILES structural formula of MAA was also obtained. Next, the potential targets of MAA in humans were predicted using the Swisstarget Prediction tool, with a screening criterion of Probability > 0. The intersections of these targets with those related to neuroinflammation were identified using the Venny tool. The STRING database was then employed to analyze the direct and indirect interactions among these targets, resulting in the creation of a protein-protein interaction (PPI) network map. The top 10 targets with the highest degree of connectivity were visualized in this network. Furthermore, the DAVID bioinformatics platform was used to perform Gene Ontology (GO) classification and pathway enrichment analysis in the Kyoto Encyclopedia of Genes and Genomes (KEGG). The results of GO terms and KEGG pathways were visualized using the Weishengxin tool.

### Statistical Analysis

All data were analyzed using (GraphPad Prism 8.3 software, USA) and are presented as mean ± SD. The normality of data distribution was confirmed using the Shapiro-Wilk test, and homogeneity of variances was verified using the Brown-Forsythe test. For comparisons across multiple groups, one-way analysis of variance (ANOVA) was performed, followed by Tukey's post-hoc test for detailed group comparisons. A *p*-value of *p < 0.05 was considered statistically significant, and **p < 0.01.

## Results

### The Inhibitory Effect of MAA on NO Production in LPS-Injured BV2 Cells

To verify that MAA can alleviate LPS-induced damage in BV2 cells, all groups except the blank control were challenged with 1 μg/ml LPS, following a pretreatment with various concentrations of MAA. As shown in Fig. 2, after treatment with MAA, the cell viability was higher compared to that of cells treated with LPS alone, indicating that MAA can mitigate LPS-induced cellular damage. Except for the 40 μM group, all other concentrations showed significant effects (*p* < 0.05), indicating a protective effect on the cells.

### The Inhibitory Effect of MAA on NO Production in LPS-Injured BV2 Cells

**Fig. 3:**
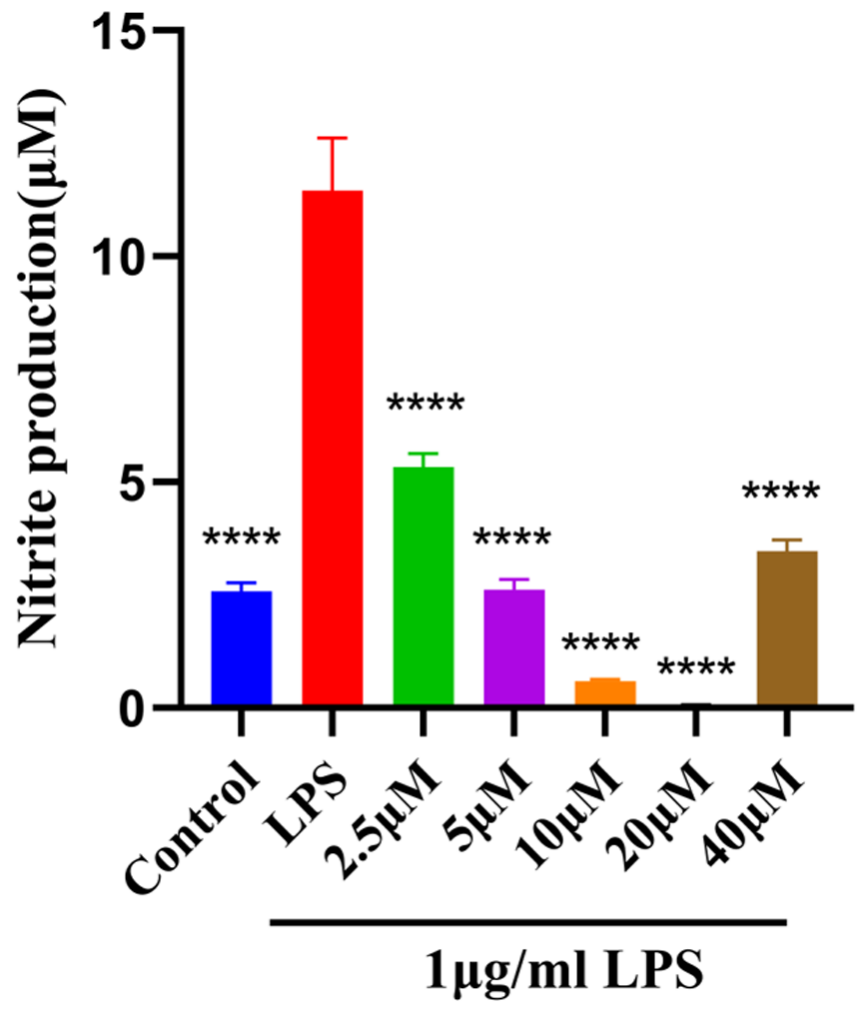
The inhibitory effects of Maackiain on NO secretion. (vs LPS group, ns (not significant difference), *p < 0.05, **p < 0.01, ***p < 0.0001, and ****p < 0.0001). Data are presented as mean ± SD.

NO is a large number of signaling-promoting inflammation molecules released within the nervous system; the amount of NO secreted by the cells functions as a crucial indicator for assessing the severity of neuroinflammation [20]. Following LPS (1 μg/ml) stimulation to all groups except the blank control, BV2 cells produced more NO than the control group, proving that the model was effective. Compared with the LPS group, MAA at all experimental concentrations significantly reduced the secretion of NO (*p* < 0.01). MAA showed the best inhibitory effect at concentrations of 20 μM, and the inhibition increased with increasing concentration at concentrations less than 20 μM (Fig. 3), indicating that at the selected concentration, MAA has potent therapeutic effects on LPS-induced neuroinflammation.

### The Inhibitory Effect of MAA on LPS-Induced ROS Production in BV2 Cells

**Fig. 4:**
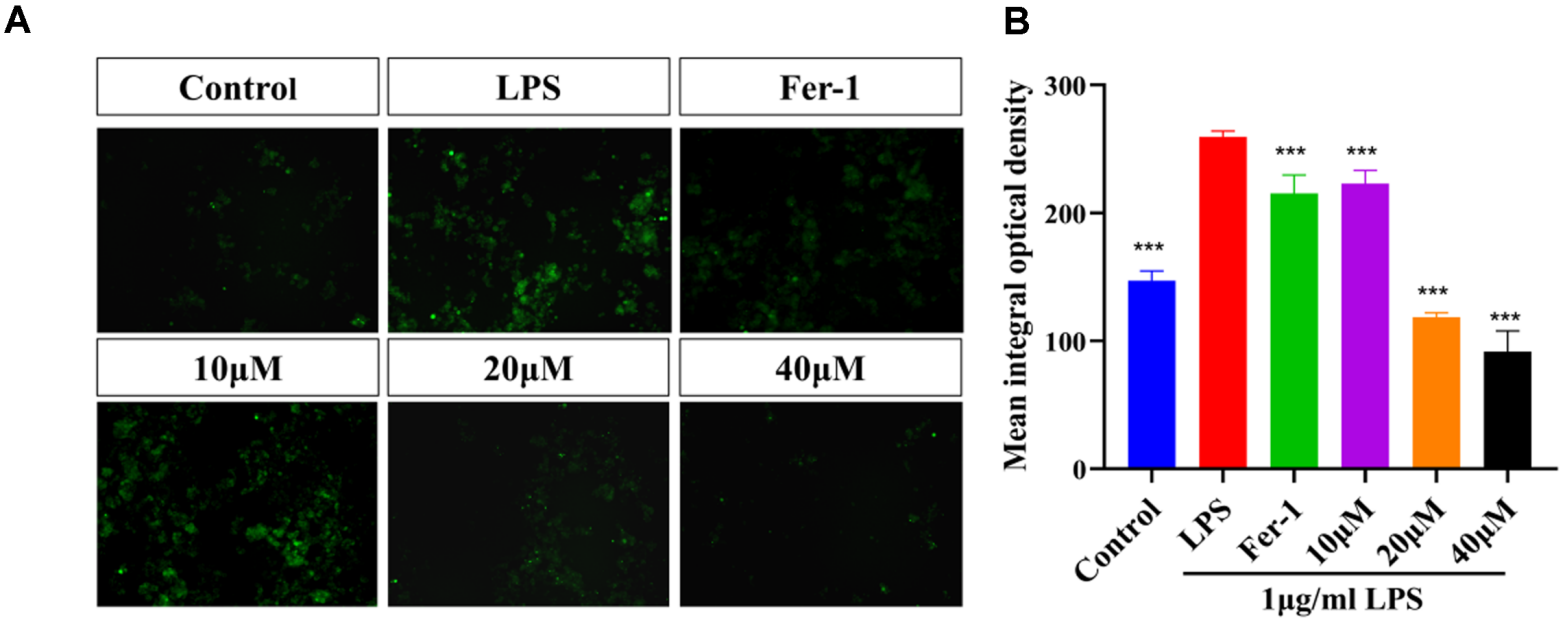
Maackiain attenuates LPS-induced intracellular ROS generation in BV2 cells. (**A**) Representative immunofluorescence images of cells stained with DCFH-DA (green) (Scale bar = 200 μm). (**B**) Quantification of relative fluorescence intensity. (vs LPS group, ns (not significant difference), *p < 0.05, **p < 0.01, ***p < 0.0001, and ****p < 0.0001). Data are presented as mean ± SD.

ROS serves as a key signal for activating multiple inflammatory pathways. As shown in Fig. 4, the experimental results demonstrated that MAA treatment at varying concentrations significantly inhibited intracellular ROS levels in BV2 cells in a concentration-dependent manner. Compared to the Control group, the 10 μM MAA treatment group already exhibited a decreasing trend in ROS levels, while the 20 μM group showed more pronounced inhibitory effects. The 40 μM MAA treatment group displayed the lowest ROS levels, indicating the strongest antioxidant efficacy. This dose-response relationship suggests that MAA may effectively scavenge intracellular ROS or suppress its generation in a concentration-dependent fashion. The 20 and 40 μM MAA treatment groups exhibited significantly lower ROS levels compared to the positive control Ferrostatin-1 group (Fer-1). These results strongly suggest that MAA may possess superior antioxidant capacity compared to classical ferroptosis inhibitors. These findings provide important clues for further investigation into the antioxidant mechanisms of MAA and its role in ferroptosis regulation.

### Effects of MAA on Fe^2+^ Levels in BV2 Cells Induced by LPS

**Fig. 5:**
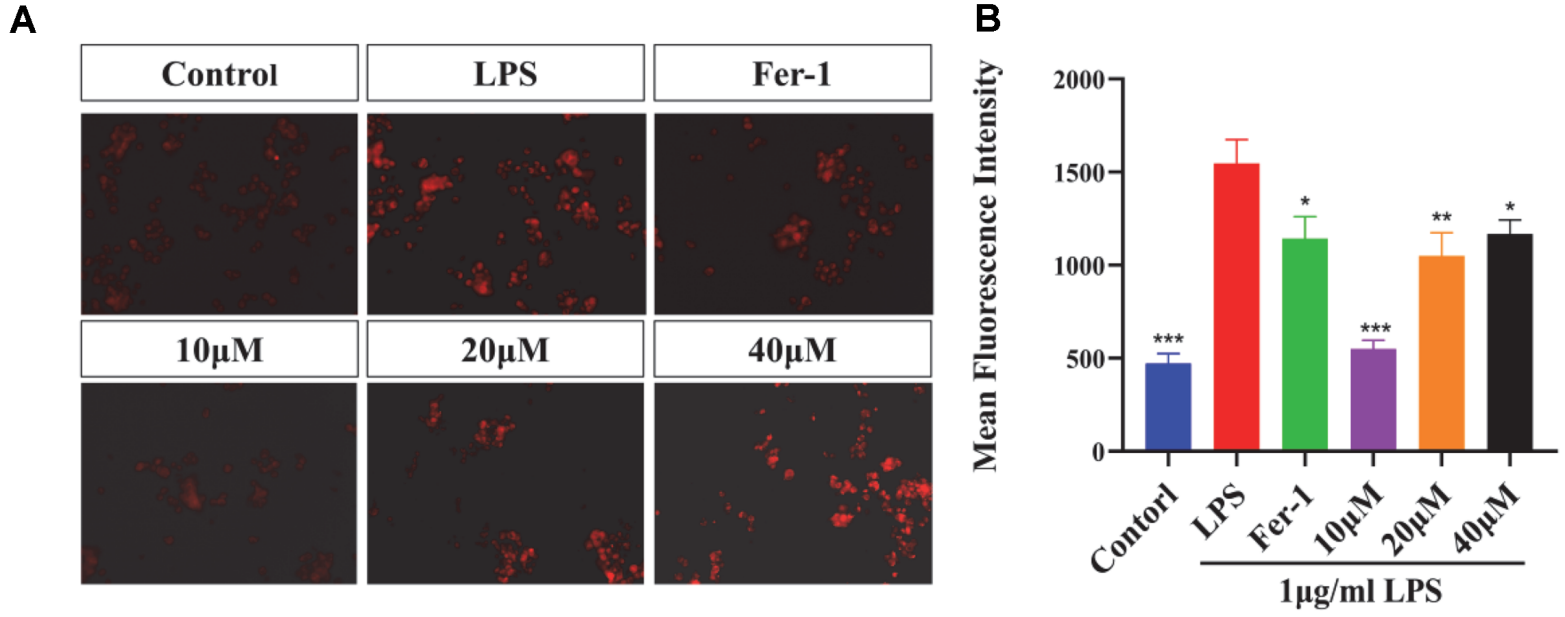
Maackiain reduces LPS-induced Fe^2+^ accumulation in BV2 cells. (**A**) Representative immunofluorescence images of cells stained with the Fe^2+^- specific probe FerroOrange (red) (Scale bar = 200μm). (**B**) Quantification of the mean optical density of FerroOrange fluorescence. (vs LPS group, ns (not significant difference), *p < 0.05, **p < 0.01, ***p < 0.0001, and ****p < 0.0001). Data are presented as mean ± SD.

The accumulation of Fe^2+^ is the core driver of ferroptosis, which generates a large amount of ROS through the Fenton reaction, leading to lipid peroxidation and cell death [21]. As shown in Fig. 5, LPS promotes the occurrence of ferroptosis by increasing the level of Fe^2+^. MAA inhibits a key step in ferroptosis by significantly reducing the level of Fe^2+^, thereby exerting an anti-inflammatory effect. These findings indicate that MAA effectively attenuates iron overload and inhibits ferroptosis, suggesting its anti-neuroinflammatory effects may be mediated through the regulation of iron homeostasis.

### Effect of MAA on Lipid Peroxidation in BV2 Cells

**Fig. 6:**
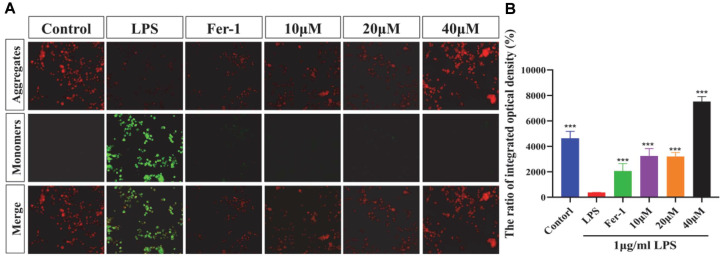
Maackiain suppresses LPS-induced lipid peroxidation in BV2 cells. (**A**) Representative immunofluorescence images of cells stained with the lipid peroxidation probe BODIPY 581/591 C_11_ (red: non-oxidized, green: oxidized) (Scale bar = 200 μm). (**B**) Quantification of lipid peroxidation, presented as the ratio of red to green (oxidized) to red (non-oxidized) fluorescence intensity. (vs LPS group, ns (not significant difference), *p < 0.05, **p < 0.01, ***p < 0.0001 and ****p < 0.0001). Data are presented as mean ± SD.

Lipid peroxidation, a core mechanism of ferroptosis, causes cell death by compromising cell membrane integrity. As shown in Fig. 6, LPS significantly increased the level of lipid peroxidation in BV2 cells (*p* < 0.001), exacerbating the occurrence of ferroptosis. All three concentrations of MAA significantly reduced the level of lipid peroxidation (*p* < 0.001), and the effect was superior to that of Fer-1. This protected the integrity of the cell membrane, thereby inhibiting ferroptosis.

### Effect of MAA on the Mitochondrial Membrane Potential in BV2 Cells

**Fig. 7:**
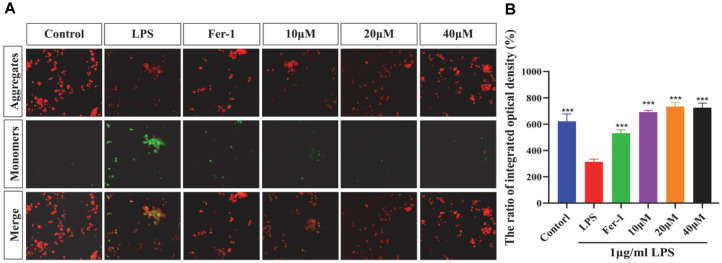
Maackiain ameliorates LPS-induced mitochondrial membrane potential loss in BV2 cells. (**A**) Representative immunofluorescence images of cells stained with JC-10 (red: J-aggregates in healthy mitochondria, green: J-monomers upon depolarization) (Scale bar = 200 μm). (**B**) Quantification of mitochondrial membrane potential (ΔΨm), presented as the ratio of red to green fluorescence intensity. (vs LPS group, ns (not significant difference), *p < 0.05, **p < 0.01, ***p < 0.0001 and ****p < 0.0001). Data are presented as mean ± SD.

The maintenance of mitochondrial membrane potential (ΔΨm) is a key factor for cellular energy metabolism and survival, and its collapse is one of the important features of ferroptosis. As shown in Fig. 7, LPS irradiation significantly decreased the red/green fluorescence ratio in BV2 cells (*p* < 0.001), indicating that LPS caused a decrease in mitochondrial membrane potential and suggesting impaired mitochondrial function. Conversely, an increase in the red/green fluorescence ratio reflects the recovery of mitochondrial membrane potential, indicating that mitochondrial function is protected. MAA significantly increased the red/green fluorescence ratio in a concentration-dependent manner (*p* < 0.001), demonstrating that MAA can effectively protect mitochondrial function and inhibit the collapse of mitochondrial membrane potential and ferroptosis.

### MAA Ameliorated LPS-Induced Cognitive and Memory Impairment

**Fig. 8:**
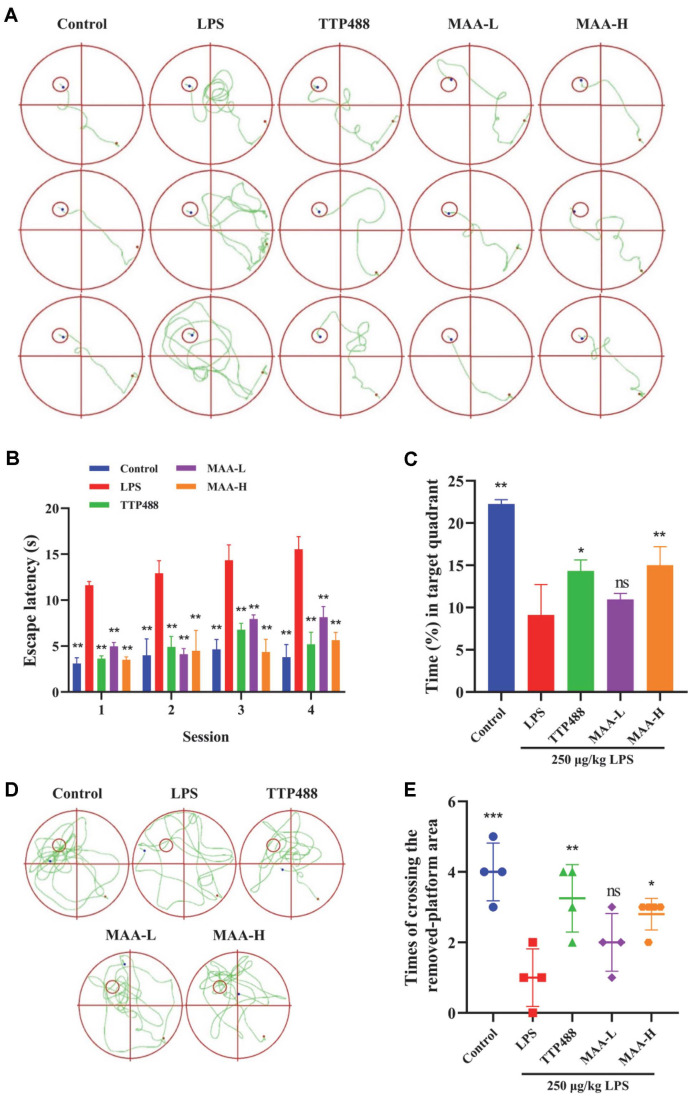
Effects of Maackiain on cognitive ability in mice with LPS-induced neuroinflammation. (**A, B**) Representative swimming paths and escape latency during the training phase. (**C, D**) Swimming paths and time spent in the target quadrant during the probe trial. (**E**) Number of platform crossings in the probe trial. (vs LPS group, ns (not significant difference), *p < 0.05, **p < 0.01, ***p < 0.0001 and ****p < 0.0001). Data are presented as mean ± SD (*n* = 10). MAA-H (50 mg/kg), MAA-L (25 mg/kg).

The water maze experiment serves primarily as a tool for evaluating cognitive impairments in animals [22]. Long-term intraperitoneal injection of LPS induces neuroinflammation, which subsequently affects the learning and memory abilities of the organism [23]. Therefore, the MWM test can be used to preliminarily evaluate the effect of MAA on improving neuroinflammation in mice. As shown in Fig. 8B, the escape latency of the mice in each group was significantly shorter than that in the LPS group (*p* < 0.01), and the movement path of the mice also verified these results Fig. 8A. The improvement effects of MAA at high concentrations were better than those of MAA at low concentrations. The longer the mice stayed in the quadrant where the previous platform was located, the better the memory ability of the mice [24]. As shown in Fig. 8C, in this part of the experiment (with the escape platform removed), mice in each group spent more time in the quadrant where the platform was originally located, compared with the LPS group, based on their memory of the platform location 5 days before training. The LPS group was significantly different from the Control group, MAA-H groups (*p* < 0.05). The improvement effect of MAA in the high concentration group was better than that in the low concentration group, and MAA-H was similar to TTP488. The movement trajectories of the mice further support these findings in Fig. 8D. Fig. 8E shows that mice in the LPS group all crossed the target platform fewer times than the remaining groups, with similar effects in TTP488, MAA-L, and MAA-H. The results of these experiments suggest that intraperitoneal injection of LPS impaired the memory ability of mice, while MAA could improve the memory function. In summary, MAA was able to ameliorate LPS injury, and MAA and TTP488 had similar ameliorative effects.

### The Neuroprotective Effects of MAA against LPS-Induced Neuronal Damage

**Fig. 9:**
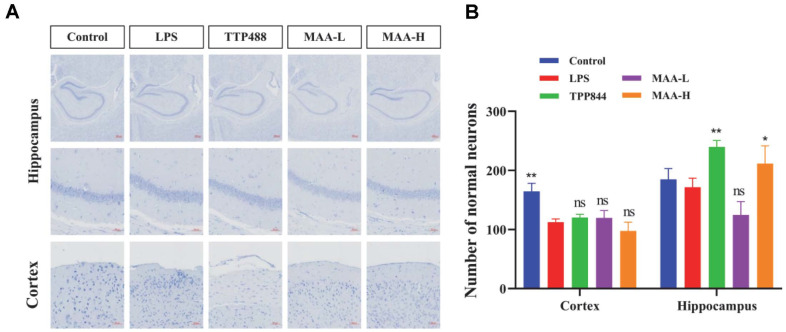
Effects of Maackiain on neurons in mice with LPS-induced neuroinflammation. (**A**) Nissl's staining of the hippocampal CA3 region and cortex (Scale bar = 20 μm). (**B**) Quantity of uninjured neurons in hippocampal CA3 and cortex. (vs LPS group, ns (not significant difference), *p < 0.05, **p < 0.01, ***p < 0.0001 and ****p < 0.0001). Data are presented as mean ± SD (*n* = 10). MAA-H (50 mg/kg), MAA-L (25 mg/kg).

Neuroinflammation leads to excessive activation of microglia, and the inflammatory mediators secreted by activated microglia can affect the development, survival, and other normal functions of neurons [25, 26]. As shown in Fig. 9, the number of normal neurons in the Control group was greater than that in the LPS group, and the number of normal neurons increased to varying degrees after MAA treatment. The MAA-H group showed a therapeutic effect in the hippocampus, while MAA-L exhibited a therapeutic effect in the cortex, but overall, it increased the number of normal neurons. This suggests that TTP488 and MAA have similar protective effects.

### Inhibitory Effects of MAA on Microglial Cell Activation

**Fig. 10:**
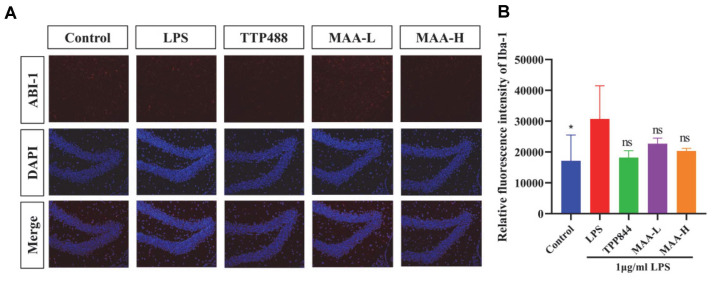
Inhibitory Effects of Maackiain on Microglial Cell Activation. (**A**) Fluorescence image of IBA-1 in mouse microglia (Scale bar = 50 μm). (**B**) The quantified assessment of IBA-1 protein analysis. (vs LPS group, ns (not significant difference), *p < 0.05, **p < 0.01, ***p < 0.0001 and ****p < 0.0001). Data are presented as mean ± SD (*n* = 10). MAA-H (50 mg/kg), MAA-L (25 mg/kg).

The activation of microglia is a major factor in the progression of neuroinflammation, and activated glial cells can release inflammatory cytokines [27]. We investigated the therapeutic power of MAA on neuroinflammation by testing its effects on LPS-induced microglia inhibition. The IBA-1 index was detected by fluorescent labeling. An increase in lBA-1 is a sign of microglial activation, and a stronger fluorescence indicates more lBA-1, representing a higher degree of microglial activation [28]. As shown in Fig. 10, the expression of IBA-1 in the LPS group demonstrated a markedly elevated value compared with the control group (*p* < 0.05), and the expression of IBA-1 in the MAA groups was decreased to varying degrees. This means that MAA has the ability to inhibit microglial activation. The effects of high concentrations of MAA were better than low concentrations.

### Effects of MAA on the Levels of Inflammatory Factors

**Fig. 11:**
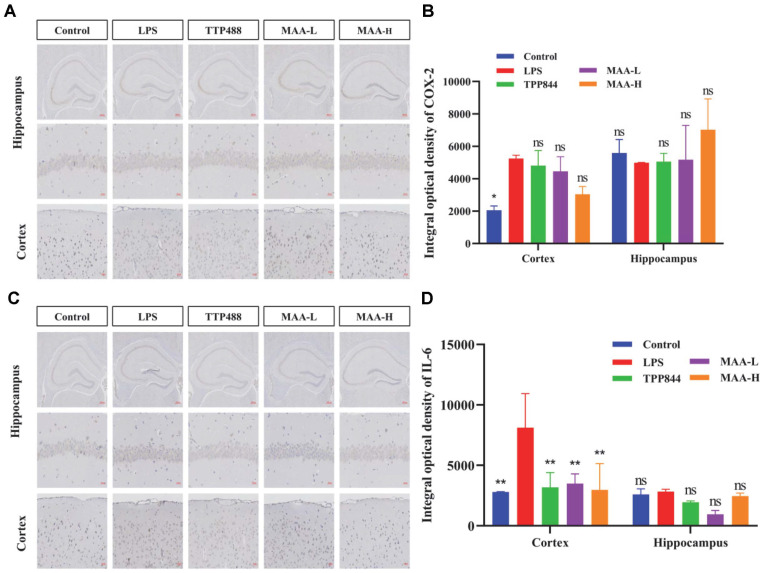
Validation of inhibition of inflammatory cytokine secretion by Maackiain. (**A, C**) Images of COX-2 and IL-6 protein in mouse brain tissue labeled by immunohistochemistry (Scale bar = 20 μm). (**B, D**) Quantitative analysis of COX-2 and IL-6 production in the mouse brain CA3 and Cortex. (vs LPS group, ns (not significant difference), *p < 0.05, **p < 0.01, ***p < 0.0001 and ****p < 0.0001). Data are presented as mean ± SD (*n* = 10). MAA-H (50 mg/kg), MAAL (25 mg/kg).

Studies have shown that microglia-derived proinflammatory cytokines are implicated in the induction of neuronal damage [29, 30]. As shown in Fig. 11B, compared with the control group, the expression of COX-2 in the cortex of the LPS group was increased, while the expression of COX-2 in the MAA group was decreased to varying degrees, and MAA-H showed the best inhibitory effect. As shown in Fig. 11D, compared with the control group, the expression of IL-6 in the cortex and hippocampus of the LPS group was increased, while the expression of IL-6 in the MAA treatment group was decreased to varying degrees. MAA-H had better inhibitory effects in the cortex. MAA inhibited the expression of COX-2 and IL-6 in the cortex and hippocampus, showing a good anti-inflammatory effect.

### Transcriptome Analysis Was Performed on the Brain Tissues of Mice Subjected to the (MWM) Task, and the Data Were Evaluated

**Fig. 12:**
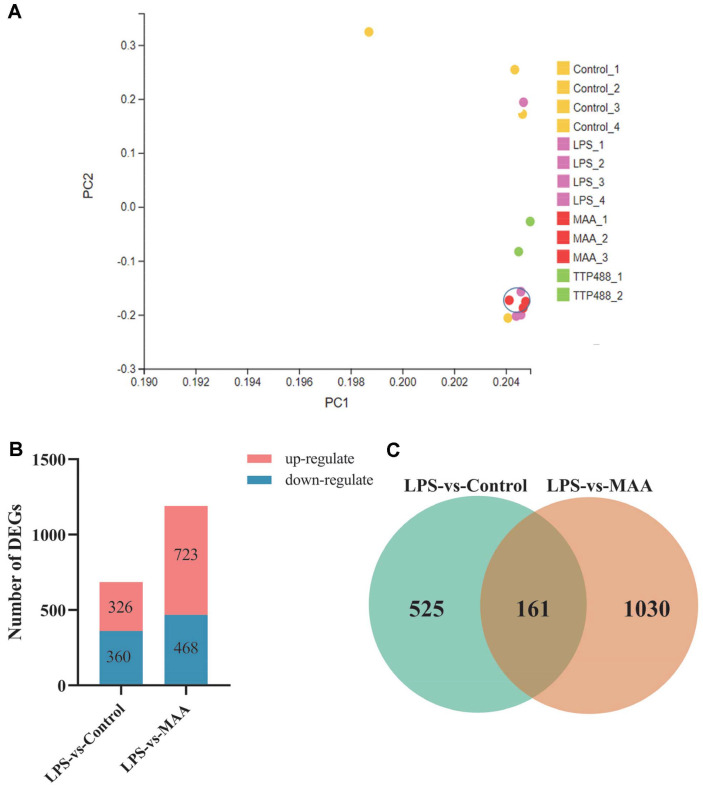
Differential Gene Expression Analysis in Neuroinflammatory Mouse Brain. (**A**) The PCA of brain tissue in neuroinflammation mice. (**C**) Expression level of differentially expressed genes. (**D**)The Venn plot of common DEGs in LPS vs Control and LPS vs MAA groups.

**Differentially expressed genes (DEGs) analysis.** Initially, we evaluated the clustering of each group using principal component analysis. As illustrated in Fig. 12A, the control group, LPS group, and MAA-treated group exhibit distinct separation between groups while maintaining tight clustering within groups, which indicates high reproducibility among samples within each group. Importantly, the observed differences between the MAA group and the TTP488 group compared to the Control group are considerably smaller than the difference observed between the LPS group and the Control group. This suggests that TTP488 and MAA demonstrate superior therapeutic effects. Subsequently, pairwise comparisons of gene expression levels were conducted to identify differentially expressed genes (DEGs). As illustrated in Fig. 12B, when comparing the LPS group with the Control group, there were 320 upregulated DEGs (red) and 360 downregulated DEGs (blue). In comparison, when the MAA group was compared to the LPS group, 723 upregulated DEGs (red) and 468 downregulated DEGs (blue) were observed. Next, Venn analysis was performed on the DEGs between the LPS group and the Control group, as well as between the MAA group and the LPS group. This analysis revealed 161 overlapping DEGs, as depicted in Fig. 12C.

**Fig. 13:**
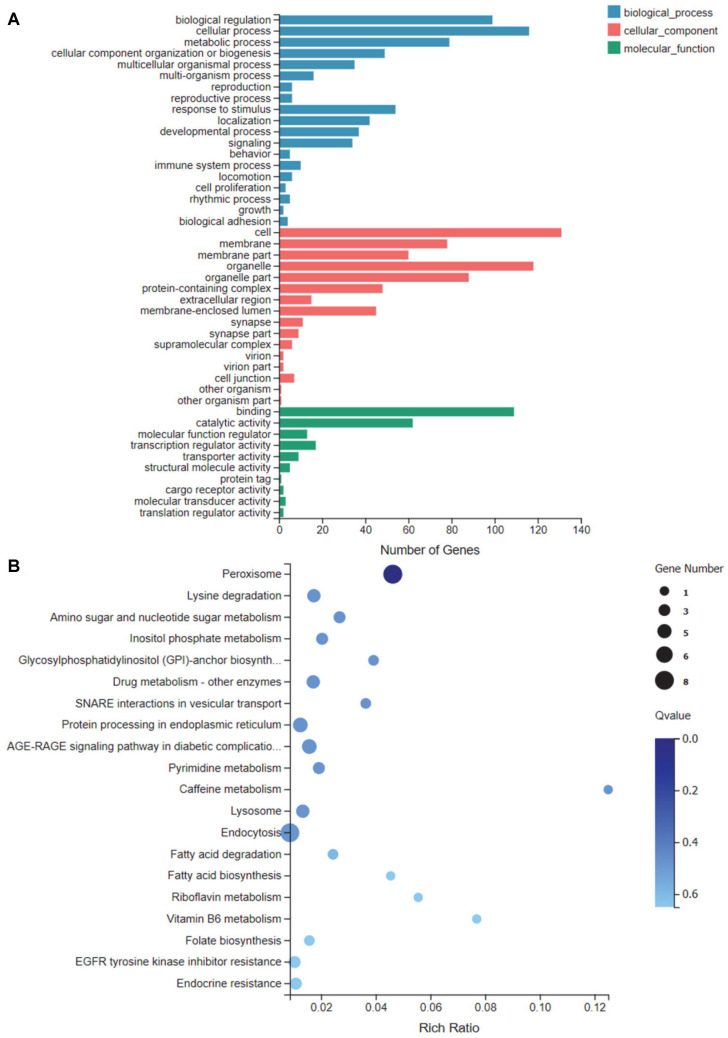
Functional Enrichment of Common DEGs. (**A**) GO enrichment analysis of common DEGs. (**B**) KEGG Pathway enrichment analysis of common DEGs.

**GO function and KEGG pathway enrichment analysis.** To elucidate the functional implications of the transcriptomic changes, we performed GO and KEGG enrichment analyses on the 161 common differentially expressed genes (DEGs). GO analysis revealed that these DEGs were primarily enriched in fundamental biological categories, including cellular processes and metabolic processes in Biological Process, cell and organelle in Cellular Component, and binding and catalytic activity in Molecular Function (Fig. 13A). To gain deeper mechanistic insights, KEGG pathway analysis was conducted, which demonstrated that the therapeutic effect of MAA was significantly associated with the modulation of several key pathways. To elucidate the underlying mechanisms, we performed KEGG pathway analysis, which indicated that MAA’s anti-neuroinflammatory activity is linked to the regulation of several pivotal pathways (Fig. 13B). These include the Peroxisome pathway, Endocytosis, Protein processing in endoplasmic reticulum, and the AGE-RAGE signaling pathway, all of which are crucial for maintaining cellular homeostasis and controlling inflammatory responses.

### Network Pharmacology

**Fig. 14:**
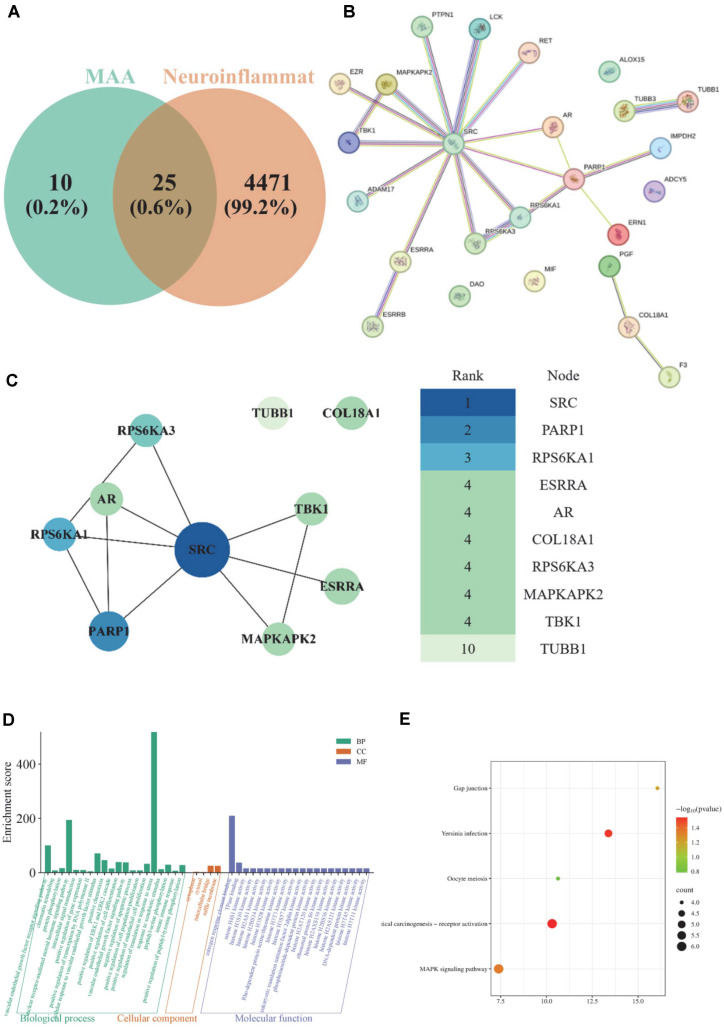
Results of network pharmacology analysis. (**A**) Enrichment of intersection targets of Maackiain and neuroinflammation-related. (**B**) Proteinmolecule interactions (PPI). (**C**) TOP10 core target enrichment. (**D**) GO enrichment diagram of Maackiain to neuroinflammation-related. (**E**) KEGG enrichment diagram of Maackiain in neuroinflammation-related.

To explore the potential targets of MAA against neuroinflammation, a network pharmacology approach was employed. We identified 25 common targets between MAA and neuroinflammation (Fig. 14A). To understand their interactions, we constructed a protein-protein interaction (PPI) network via the STRING database, which comprised 25 nodes and 57 edges (Fig. 14B). The top 10 core targets were then identified based on degree values and visualized in Cytoscape (Fig. 14C). As shown, the most influential targets were SRC, PARP1, RPS6KA1, ESRRA, AR, COL18A1, RPS6KA3, MAPKAPK2, TBK1, and TUBB1. The size and color of the nodes reflect their importance, highlighting these molecules as potential pivotal hubs for MAA’s therapeutic action.

Subsequently, Gene Ontology (GO) and Kyoto Encyclopedia of Genes and Genomes (KEGG) enrichment analyses were performed on the 25 intersecting targets. As shown in Fig. 14D, the GO analysis revealed that the most significantly enriched functions were within the Biological Process and Molecular Function categories. These terms indicate that MAA’s therapeutic effects against neuroinflammation are associated with biological processes such as regulation of translation in response to stress, nuclear receptor-mediated steroid hormone signaling pathway, estrogen response element binding, and vascular endothelial growth factor receptor signaling pathway. Furthermore, KEGG analysis was conducted to identify the key pathways involved. As depicted in Fig. 14E, the primary pathways through which MAA intervenes in neuroinflammation include chemical carcinogenesis-receptor activation, Yersinia infection, MAPK signaling pathway, gap junction, and oocyte meiosis.

## Discussion

Neuroinflammation is a common feature of various neurodegenerative diseases; therefore, targeting it has emerged as a promising therapeutic strategy [31]. Fe^2+^ catalyzes the production of ROS via the Fenton reaction, which in turn attacks polyunsaturated fatty acids in the cell membrane, initiating a lipid peroxidation chain reaction [32]. This process, in concert with excessive ROS, disrupts mitochondrial structure and function, leading to the depolarization of the mitochondrial membrane potential. Consequently, the dysfunctional mitochondria become a secondary source of ROS, creating a self-amplifying cycle of damage [33]. This cascade of events constitutes the core biochemical basis of ferroptosis [34]. Notably, a profound interplay exists between ferroptosis and neuroinflammation. Lipid peroxidation products and Fe^2+^ released from ferroptotic cells can act as damage-associated molecular patterns, activating microglia and prompting them to release pro-inflammatory cytokines such as IL-6 and TNF-α, thereby initiating and exacerbating neuroinflammation [35]. Conversely, the ROS and inflammatory signals enriched in the neuroinflammatory microenvironment can further suppress the cellular antioxidant defense system, thereby aggravating ferroptosis and establishing a “ferroptosis-neuroinflammation” vicious cycle [36]. This mutually reinforcing pathological mechanism plays a pivotal role in the progression of neurodegenerative diseases, including Alzheimer’s and Parkinson’s disease. This study demonstrates that MAA effectively reduces intracellular ROS levels, inhibits the excessive accumulation of Fe^2+^, and alleviates lipid peroxidation, while concurrently restoring mitochondrial membrane potential. These findings suggest that MAA can not only directly suppress ferroptosis but may also indirectly inhibit the overactivation of microglia by reducing the release of damage-associated molecular patterns (DAMPs), thereby disrupting the ferroptosis-neuroinflammation vicious cycle. This dual mechanism of action may be key to the therapeutic potential of MAA in treating neuroinflammation.

The activation of microglia is closely associated with neuroinflammation. LPS stimulates microglia to release pro-inflammatory mediators, and ROS can induce neuronal damage, while IL-6 and COX-2 are neurotoxic to microglia [37]. In the hippocampus and cerebral cortex, neuronal injury leads to cognitive dysfunction [38]. IL-6 and COX-2 are key molecules in neuroinflammation and apoptosis, and their upregulated expression is a hallmark of the inflammatory state [39, 40]. This study found that MAA ameliorates LPS-induced learning and memory deficits by inhibiting microglial activation and the production of pro-inflammatory cytokines. Concurrently, it reduces inflammation and maintains neuronal homeostasis by modulating IL-6 and COX-2. These findings prompted us to further investigate the underlying anti-neuroinflammatory mechanisms of MAA.

Through transcriptomic analysis, we discovered that MAA alleviates neuroinflammation by altering pathways such as the Peroxisome pathway, Endocytosis, Protein processing in endoplasmic reticulum, and the AGE-RAGE signaling pathway. The peroxisome plays a pivotal role in regulating ROS metabolism, lipid peroxidation, and inflammatory signaling. Its dysfunction can lead to exacerbated oxidative stress, promoting microglial activation and the progression of neuroinflammation [41]. Endocytosis is involved in the recognition and clearance of damage-associated molecular patterns (DAMPs) by microglia, as well as the recycling and signaling regulation of inflammatory receptors. Abnormalities in endocytic function can result in sustained activation of inflammatory signals, exacerbating neuroinflammation and neurodegenerative pathologies [42]. Endoplasmic reticulum (ER) stress and the unfolded protein response (UPR) are critical drivers of neuroinflammation. Persistent ER stress can activate inflammatory pathways such as NF-κB, promoting the release of pro-inflammatory factors, including IL-6 and TNF-α, leading to neuronal damage [43]. The AGE-RAGE signaling pathway is a pivotal inflammatory axis in diabetes, aging, and neurodegenerative diseases. RAGE activation can trigger ROS bursts, NF-κB activation, and high expression of pro-inflammatory factors, and is closely associated with neuroinflammation in conditions such as Alzheimer’s disease [44].

To predict other potential targets of MAA in treating neuroinflammation, a network pharmacology analysis was conducted. The results suggested that SRC, PARP1, RPS6KA1, ESRRA, AR, COL18A1, RPS6KA3, MAPKAPK2, TBK1, and TUBB1 may be the core targets. GO and KEGG enrichment analysis of the common targets revealed that the biological functions involved in MAA’s treatment of neuroinflammation include regulation of translation in response to stress, nuclear receptor-mediated steroid hormone signaling pathway, estrogen response element binding, and vascular endothelial growth factor receptor signaling pathway. The primary mechanisms involved include chemical carcinogenesis-receptor activation, Yersinia infection, the MAPK signaling pathway, gap junction, and oocyte meiosis. Among these, pathogens can induce a strong neuroinflammatory response by activating pro-inflammatory pathways such as TLR/NF-κB through their outer membrane proteins and virulence factors, leading to microglial activation, IL-6/TNF-α release, and neuronal damage. This is a significant trigger for encephalitis and neurodegenerative diseases [45]. MAPK (especially p38, JNK, and ERK) is a key hub that regulates microglial activation, inflammatory factor release, oxidative stress, and neuronal apoptosis. Overactivation of MAPK can drive neuroinflammation and various neurodegenerative pathologies [46]. Gap junctions (such as Cx43 and Cx32) regulate intercellular communication and the neuron-glia network. Dysregulation of gap junctions can amplify neuroinflammation, disrupt the blood-brain barrier, and exacerbate neuronal damage, representing a key mechanism in various encephalopathies and neurodegenerative diseases [47], which could help explain the anti-neuroinflammatory mechanism of MAA.

In summary, this study demonstrates that MAA exerts anti-neuroinflammatory effects through a multi-target and multi-pathway approach. The results show that MAA effectively attenuates LPS-induced neuroinflammation by directly inhibiting ferroptosis—reducing intracellular accumulation of ROS and Fe^2+^, suppressing lipid peroxidation, and restoring mitochondrial membrane potential to inhibit mitochondrial damage. *In vivo*, MAA significantly improves LPS-induced cognitive impairment, protects neurons in the hippocampus and cortex, and inhibits the production of key pro-inflammatory mediators such as IL-6 and COX-2 by suppressing microglial activation. Transcriptomic and network pharmacology analyses indicate that MAA regulates key pathways, including the peroxisome pathway, endocytosis, protein processing in the endoplasmic reticulum, and the AGE-R signaling pathway. Furthermore, core targets such as SRC, PARP1, MAPKAPK2, and RPS6KA3 were predicted, suggesting that the therapeutic effects of MAA may be mediated through a complex regulatory network involving MAPK signaling, gap junction communication, and receptor-mediated inflammatory responses. Overall, this study provides a preclinical evaluation of the anti-neuroinflammatory properties of MAA, and these data highlight its potential as a promising therapeutic agent for neuroinflammation and related neurodegenerative diseases. However, the results of transcriptomic and network pharmacology analyses have not been fully validated by empirical studies. Future research should prioritize validating the accuracy of the identified pathways in transcriptomics and network pharmacology analyses across both *in vitro* and *in vivo* models. This will help to better elucidate the molecular mechanisms underlying MAA's anti-neuroinflammatory effects.

## Conclusion

In summary, this study demonstrates that MAA exerts anti-neuroinflammatory effects through a multi-target and multi-pathway approach. The results show that MAA effectively attenuates LPS-induced neuroinflammation by directly inhibiting ferroptosis—reducing intracellular accumulation of ROS and Fe^2+^, suppressing lipid peroxidation, and restoring mitochondrial membrane potential to inhibit mitochondrial damage. *In vivo*, MAA significantly improves LPS-induced cognitive impairment, protects neurons in the hippocampus and cortex, and inhibits the production of key pro-inflammatory mediators such as IL-6 and COX-2 by suppressing microglial activation. Transcriptomic and network pharmacology analyses indicate that MAA regulates key pathways, including the peroxisome pathway, endocytosis, protein processing in the endoplasmic reticulum, and the AGE-R signaling pathway. Furthermore, core targets such as SRC, PARP1, MAPKAPK2, and RPS6KA3 were predicted, suggesting that the therapeutic effects of MAA may be mediated through a complex regulatory network involving MAPK signaling, gap junction communication, and receptor-mediated inflammatory responses. Overall, this study provides a preclinical evaluation of the anti-neuroinflammatory properties of MAA, and these data highlight its potential as a promising therapeutic agent for neuroinflammation and related neurodegenerative diseases.

## Figures and Tables

**Fig. 1 F1:**
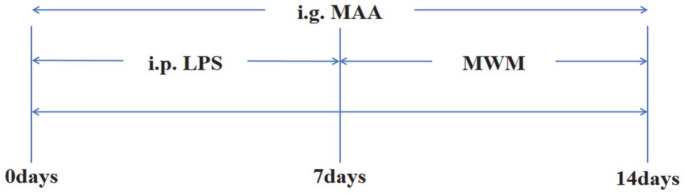
Flow chart of the experiment.
